# Serum and Lymphocytic Neurotrophins Profiles in Systemic Lupus Erythematosus: a Case-Control Study

**DOI:** 10.1371/journal.pone.0079414

**Published:** 2013-11-01

**Authors:** Anne-Laure Fauchais, Marie-Claude Lise, Pierre Marget, François-Xavier Lapeybie, Holy Bezanahary, Clothilde Martel, Stéphanie Dumonteil, Agnès Sparsa, Fabrice Lalloué, Kim Ly, Marie Essig, Elisabeth Vidal, Marie-Odile Jauberteau

**Affiliations:** 1 Limoges University Hospital, Equipe Accueil 3842-Clinical Immunology Laboratory, Fr GEIST, Limoges, France; 2 Department of Internal Medicine, Limoges University Hospital, Limoges, France; 3 Department of Dermatology, Limoges University Hospital, Limoges, France; 4 Department of Nephrology, Limoges University Hospital, Limoges, France; University of Düsseldorf, Germany

## Abstract

**Background:**

Neurotrophins play a central role in the development and maintenance of the nervous system. However, neurotrophins can also modulate B and T cell proliferation and activation, especially via autocrine loops. We hypothesized that both serum and lymphocytic neurotrophin levels may be deregulated in systemic Lupus erythematosus (SLE) and may reflect clinical symptoms of the disease.

**Methods:**

Neurotrophins in the serum (ELISA tests) and lymphocytes (flow cytometry) were measured in 26 SLE patients and 26 control subjects. Th1 (interferon-γ) and Th2 (IL-10) profiles and serum concentration of BAFF were assessed by ELISA in the SLE and control subjects.

**Findings:**

We have demonstrated that both NGF and BDNF serum levels are higher in SLE patients than healthy controls (*p*=0.003 and *p<0.001*), independently of Th1 or Th2 profiles. Enhanced serum NT-3 levels (*p*=0.003) were only found in severe lupus flares (i.e. SLEDAI ≥ 10) and significantly correlated with complement activation (decreased CH 50, Γ=-0.28, *p*=0.03). Furthermore, there was a negative correlation between serum NGF levels and the number of circulating T regulatory cells (Γ=0.48, *p*=0.01). In circulating B cells, production of both NGF and BDNF was greater in SLE patients than in healthy controls. In particular, the number of NGF-secreting B cells correlated with decreased complement levels (*p*=0.05). One month after SLE flare treatment, BDNF levels decreased; in contrast, NGF and NT-3 levels remained unchanged.

**Conclusion:**

This study demonstrates that serum and B cell levels of both NGF and BDNF are increased in SLE, suggesting that the neurotrophin production pathway is deregulated in this disease. These results must be confirmed in a larger study with naive SLE patients, in order to avoid the potential confounding influence of prior immune-modulating treatments on neurotrophin levels.

## Introduction

 The neurotrophins (NT) are a family of proteins comprising nerve growth factor (NGF), brain-derived neurotrophic factor (BDNF), Neurotrophin-3 (NT-3), and NT-4/5, initially identified through their crucial function in nervous system development, growth control, and neuron and astrocyte apoptosis [1]. However, the effects of these growth factors are much more broad, and extend to a wide range of cell types, including immune cells. Indeed, growing evidence suggests that NGF, BDNF, and NT-3 participate in inflammatory responses, including the modulating and regulating immune function in inflammatory and autoimmune diseases [1]. 

 Neurotrophin function in immune regulation has been assessed in several reports that demonstrate that immune cells both secrete and are targets of the three major NTs (NGF, BDNF and NT3). Indeed, after activation, B cells, plasmocytes and T cells express NT receptors (TrkA, TrkB, TrkC and p75^NTR^) and produce functional NGF, BDNF, and NT-3, which is involved in lymphocyte maturation, proliferation, and activation [2-6]. Concerning B lymphocytes, NGF is secreted during B cell activation, which triggers their proliferation and differentiation into plasma cells [7-10]. Immunoglobulin secretion (IgG and IgM or IgE) is enhanced by NGF or BDNF [8-11]. In addition, BDNF also plays an important role in B cell development. This was demonstrated in BDNF^-/-^ mice, which show a developmental arrest in B cell maturation at the pre-BII stage [12]. Lastly, both NGF and BDNF are autocrine factors for mature B and plasma cell survival [2,13], whereas this function for NT-3 has only been reported during inflammatory conditions [4]. 

 NTs also exert important functions in T lymphocytes: they may promote T cell activation of a Th2 or Th1 profile. Indeed, NGF enhances Th2 cell proliferation and modulates T-cell-dependent antibody synthesis and T cell production of gamma-interferon (INF-γ) [10,14-18]. Moreover, CD40L, interleukin-1β (IL-1β), IL-4 and *tumor necrosis factor-alpha* (TNF-α) upregulate production of NGF in lymphocytes [19]. In contrast, INF-γ decreases NT synthesis [20]. Th2 cytokines can also upregulate BDNF production in immune cells [21]. NT3 synthesis is enhanced in Th1-activated human lymphocytes [22]. Thus, these data support a potential crosstalk between NTs and Th1 and Th2 cytokine profiles during the inflammatory response.

 Data on immune cell expression of NT-4/5 are sparse. NT-4/5 is expressed by 25% of human circulating peripheral blood mononuclear cells (PBMC), activated human T cells, and murine alveolar macrophages [23-25]. However, the function of this neuropeptide, known to interact with the TrkB receptor in neural cells, remains unknown in immune cells. 

 The relationship between NT-secreting immune cells and the resulting tissue damage has been evaluated in some chronic inflammatory-autoimmune diseases. During rheumatoid or psoriasis arthritis, synovial CD3+ T lymphocytes and monocytes/macrophages produce high levels of NGF, which enhance both fibroblast-like cell proliferation and synovial T cell activation via TrkA ligation and Akt phosphorylation [26,27]. In sarcoidosis, epithelioid and multinucleated giant cells of the granuloma, alveolar macrophages and T cells produce NGF, BDNF and NT-3 [28,29]. CD4 and CD8 NT expression correlates with the sarcoidosis radiological damage index [29]. In contrast, in Crohn’s disease, local secretion of NT, especially NGF and BDNF by mast cells, reduces enteric glia cell apoptosis induced by pro-inflammatory cytokines [30,31]. 

 Together, these findings suggest that NT, excessively produced by immune cells in autoimmune diseases, may participate in disease progression by modulating both immune cell function and tissue lesions. 

 Based on this foundational data, other studies have evaluated serum NT levels in various autoimmune and pro-inflammatory diseases. However, these reports have mainly dealt with NGF [32]. Indeed, serum NGF concentrations are increased in juvenile arthritis [33], Kawasaki disease [34], Behçet’s disease [35], systemic sclerosis [36,37] and primary Sjögren’s syndrome [32,38]. Increased BDNF levels in sera have also been reported in primary Sjögren’s syndrome, which correlates with systemic activity and B and T cell activation [38]. In contrast, serum BDNF levels are decreased in systemic sclerosis, reflecting the vascular aspect of the disease [36]. It has also been reported that NT-3 is upregulated only in autoimmune diseases strongly affecting the joints [36,38]. Serum NT-4/5 levels are upregulated in mood disorders but have not been yet evaluated in autoimmune disease [39]. 

 There is little data on lymphocytic NT expression in human inflammatory disease. BDNF-secreting T cells are reduced in untreated multiple sclerosis patients and increased after interferon beta treatment [40], while NGF, NT-3 and NT-4 production by PBMCs in multiple sclerosis patients is enhanced in the post-relapse phase [41]. In contrast, BDNF production is unchanged in B and T cells in systemic sclerosis patients compared to healthy controls [36].

 In SLE, few studies have focused on NT expression and its relationship to disease activity. In NZB/W mice, serum NGF concentrations are significantly increased, correlating with an accumulation of NGF-containing cells in the kidney and spleen [42]. NGF levels are higher in the sera of SLE patients than healthy controls [43,44] and reflect systemic activity of the disease as assessed by the SLEDAI (SLE Disease Activity Index) score [44]. However, reports on serum BDNF concentration in SLE are contradictory and limited to neuropsychiatric forms of the disease. Though serum BDNF levels are decreased in neuro-SLE according to one case report [45], they are increased in two other studies [45,46]. 

The aim of the present study was to evaluate serum and lymphocytic levels of NGF, BDNF and NT-3 in SLE patients and identify their relation to clinical features (systemic activity assessed by SLEDAI score, joint, skin, neurological and kidney involvement, vasculitis), SLE-related immunological activity (anti-native DNA antibodies, complement activation via CH 50, C3 and C4 levels), and anti-phospholipid antibodies. Furthermore, we evaluated B cell activation parameters that could be modulated by SLE (serum BAFF levels and autoantibody production) and their association with enhanced levels of NT in sera [36,38]. Additionally, we analyzed the cytokine profiles and T-regulatory cell population that could be modified by SLE activity [47]. IL-10 and IFN-γ, two cytokines belonging to the TH2 and TH1 profile, respectively, were measured in sera from controls and patients. Several studies have previously demonstrated that IL-10 levels in sera are significantly higher in untreated SLE patients than in healthy controls [48] and strongly reflect SLE activity [49]. Their dynamics are closely linked to those of autoantibody synthesis [50,51]. Strikingly, circulating IL-10 levels decrease after treatment and correlate with a change in the SLEDAI score, indicating that sera IL-10 level is a biological marker of SLE activity [52]. Moreover, the evaluation of IL-10 levels in SLE is also supported by the direct effect of IL-10 on NGF secretion in a murine astrocyte model [20]. Likewise, a relationship between IFN-γ and BDNF cell activation has been established in microglial cells, in that BDNF inhibits IFN-γ-induced activation [53].

*Considering that no reported experimental data support the involvement of NT4/5 in B and T cell activation or autoimmune disease, this study mainly focused on the relationship between serum levels, B-lymphocyte expression of NGF, BDNF and NT-3, and SLE activity.*

## Materials and Methods

### Patients and control population

 Twenty-six successive SLE patients, including 24 women (median age 44±12 years), were included in a one-year cross-sectional study (2011) in Limoges University Hospital [54]. All participants fulfilled the revised American College of Rheumatology (ACR) criteria for SLE. Disease activity was evaluated using the SLEDAI score [55]. Neurological complications were ruled out by clinical examination combined with normal brain MRI (n=11) and brain 18-F-fluorodeoxyglucose positron emission tomography (n=2).

 Patients with neoplastic disorders or depression were excluded in order to avoid confounding effects on serum NT levels [56]. 

 The control population consisted of 26 healthy age- and sex-matched volunteers. Patients were excluded if they were pregnant, under the age of 18, or unable to give valid consent. The “NeuroLED” study received ethical approval from The Limoges University Hospital Research Ethics Committee (N°I06023), and was carried out in accordance with the Helsinki Declaration. Written consent was obtained from all patients and control subjects.

### Clinical features

The clinical features of SLE present at the time of blood sampling were cutaneous (n=22, 85%), articular (n=21, 81%), renal (n=4, 16%), neurological (n=2, 8%) and pleural (n=1, 4%), as well as pericarditis (n=1) and vasculitis (n=9; Table 1). The mean SLEDAI score was 7.2 ± 4.1 (range 4-20). Six patients (23%) presented a severe systemic SLE flare defined by a mean SLEDAI score over 9 (Table 1). Six patients (23%) presented an associated anti-phospholipid syndrome and 7 presented secondary Sjögren’s syndrome (27%, Table 1).

**Table 1:** Clinical and biological features of the SLE patient group.

Patient/age/gender	Clinical manifestations	SLEDAI	AAPS, SS	Treatment	NGF (pg/mL)	BDNF (pg/mL)	NT3 (pg/mL)	APL, antiß2GP1	CH 50	Anti-nuclear Ab / nDNA
1/41/F	Skin	4	1/1	HCQ / 0	559.7	511.1	2546.2	0/0	700	1/320 - 18
2/30/F	Skin, Art	6	0/0	HCQ / 1	425.9	433.3	1788.5	0/1	370	1/640 - 200
3/33/F	Skin, Art	4	0/0	HCQ, P, MMF / 1	395.7	383.8	2876.2	0/0	750	1/1280- 48
4/40/F	Skin, Art	4	0/0	HCQ, P / 0	408.1	434.3	3112.4	0/0	750	1/1280 - 114
5/54/M	Art	6	0/1	- / 0	348.3	631	4378.8	0/0	750	1/640 - 87
6/66/F	Skin, Art, P	10	0/0	HCQ, P / 0	471.4	749.1	4414	0/0	750	1/160 - 15
7/55/F	Skin, Art	6	0/0	HCQ / 0	364.5	596.7	1568.5	1/0	190	1/1280 - 61
8/43/F	Skin, Art, Ren, V	6	0/1	HCQ, P / 1	520.7	567.7	4204.2	1/1	650	1/1280 - 200
9/30/F	Skin, V	8	0/1	- / 0	480.2	567.7	1247.8	0/0	270	1/1280 - 12
10/40/F	Skin, Art	4	0/0	HCQ / 0	404.4	725.0	1846.5	0/0	750	-
11/24/F	Skin, Art, Ren, M	8	0/0	HCQ, P / 1	396.9	799.8	2139.7	0/0	570	1/1280 - 113
12/25/F	Skin, Art, P	4	0/0	HCQ, P / 1	404.7	665.1	2307.8	0/0	570	1/640 - 12
13/50/F	Art, Ren	8	0/0	HCQ, P / 1	411.7	651.4	4261.2	0/0	570	1/1280 - 200
14/48/F	Skin, Art	4	0/0	P / 1	443.9	549.1	2717.5	0/0	240	1/1280 - 119
15/47/F	Art, Ren, CNS	20	0/0	P / 1	420.5	556.3	3180.1	0/0	570	1/1280 - 200
16/45/F	Skin, V	5	1/0	HCQ, P / 0	418.3	617	1753.2	1/0	430	1/1280 - 320
17/30/F	Skin	4	0/0	HCQ / 1	440.4	667	2098.2	0/0	380	1/320
18/59/F	Skin, V	8	0/0	- / 1	372.8	694.1	1163	1/0	650	1/320
19/39/F	Skin, Art, Ren, V	10	1/0	HCQ, P, Aza / 1	309.3	627.1	3619.2	1/0	600	1/1280 - 58
20/77/F	Skin, Art, V	8	1/1	- / 1	263.4	584.9	1618.5	1/0	500	1/320
21/44/F	Skin, Art, V	12	0/1	HCQ / 1	426.8	548.1	3425.5	0/0	320	1/640
22/38/F	Skin, Art, V	12	1/0	HCQ, P / 1	486.4	581.7	5946.3	1/0	330	1/1280 - 12
23/31/M	Skin, Art, CNS, V	15	0/0	HCQ / 1	442.8	613.2	4379.6	1/1	210	-
24/55/F	Art	4	1/0	HCQ, P, Aza / 1	522.7	358.2	3682.7	1/1	570	1/1280 - 12
25/53/F	Skin, Art	4	0/1	HCQ / 0	375.7	781	3928.1	0/0	700	1/320
26/50/F	Skin, Art, Dig	4	0/0	HCQ, P, MMF / 0	520.7	472.3	1436.5	0/0	570	1/1280 - 200

Clinical manifestations: {skin manifestations (Skin), articular (Art), renal (Ren), central nervous system (CNS), muscular (M), and digestive (Dig) involvements, pleural effusion and pericarditis (P), vasculitis (V)}, SLEDAI score, associated antiphospholipid syndrome (AAPS), secondary Sjögren’s syndrome (SS) associated with SLE. Immunomodulating treatment: {prednisone (P), hydroxychloroquine (HCQ), mycophenolate mofetil (MMF), azathioprine (Aza)} was increased (1) or not (0) after sampling. Serum dosages of NGF, BDNF and NT3 (pg/mL) and immunological profiles including anti-phospholipid (APL) and anti- native DNA antibodies Ab (nDNAn) are summarized.

Out of the 26 patients, 14 (54 %) were treated with glucocorticoids (mean 18.1 ± 22.2 mg/day), 20 (77%) with anti-malarial drugs, and 4 (16%) with immunosuppressants (azathioprine n=2, mycophenolate mofetil to maintain remission of a previous renal flare n=2; Table 1). 

Treatment was increased directly after the blood sampling for systemic flare in 61% of cases (n=16) with the initiation of hydroxychloroquine (n=5, 19%), glucocorticoid (n=7, 27%) or immunosuppressants (n=7, 27%), or with an increase of previous corticosteroid dosages alone (n=3, 11%; Table 1). 

Another blood sample for serum NT levels was taken one month after the systemic flare in 9 cases.

### Additional assessment of quality of life

 Patients completed the Medical Outcomes Study, a 36-item short form health survey (SF-36), independently of their visit to the physician [57]. The questionnaires were returned to the research assistant. 

### Measurement of autoantibodies and neurotrophin levels

 Antinuclear antibodies were characterized by immunofluorescence in HEp2 cells (The Binding Site, Saint Egrève, France) and anti-native DNA (nDNA) antibodies by ELISA (Phadia, Saint-Quentin Yvelines, France). Anti-cardiolipin and anti-β2 Glycoprotein 1 (β2-GP1) antibodies were measured by ELISA (Ingen, Chilly Mazarin, France).

 Serum NGF, BDNF and NT-3 levels were measured using commercial ELISA kits according to the manufacturer’s instructions (NGF E_max_^®^ ELISA, BDNF E_max_^®^ ELISA, NT-3 ELISA, Promega, Charbonnières, France). All assays were performed in duplicate and the data are presented as pg/mL. Detection limits were 15 pg/mL for BDNF and 4 pg/mL for NT-3 and NGF. 

### B cell activation analysis and determination of Th1 and Th2 profiles

Serum BAFF, INF-γ (Th1) and IL-10 (Th2) levels were measured with an ELISA kit according to the manufacturer’s instructions (Quantikine^®^ Human Immunoassay R&D system, Lille, France). All assays were performed in duplicate and the data are presented as pg/mL. Detection limits were 4 pg/mL for BAFF and 2 pg/mL for INF-γ and IL-10.

### Determination of neurotrophin expression by B and T cells

Expression of intracellular NGF, BDNF and NT-3 in T and B lymphocytes was assessed by flow cytometry. Whole blood cells were stained with either phycoerythrin (PE)-cyanin (Cy) 7-conjugated anti-CD3 or anti-CD19 antibodies for 15 min at room temperature. After lysing the red blood cells (Immunoprep, Beckman Coulter, France), white blood cells were fixed, permeabilized (Intraprep, Beckman Coulter) and incubated at room temperature for 30 min with rabbit anti-NGF, anti-BDNF and anti-NT-3 antibodies (all 1/100; Santa Cruz Biotechnology, France) in Phosphate-Buffered Saline (PBS) containing 1% Bovine Serum Albumin. After two washes in PBS, antibodies were detected with Alexa Fluor 488-conjugated goat anti-rabbit IgG antibodies (10 μg/mL; Invitrogen, France) for 30 min at 4°C. Cells stained with rabbit isotypic immunoglobulins (Santa Cruz Biotechnology, France) were used as controls to determine background and positive result thresholds. After washing twice in PBS, cells were suspended in PBS and analyzed with a flow cytometer (FacsCanto^TM^ II, Becton Dickinson, Le Pont-de Claix, France).

### Quantitative analysis of circulating T regulatory cells

Cells were stained with Cy7-conjugated anti-CD3, FITC-conjugated anti-CD4, Cy5-anti-CD25 (Beckman Coulter), and PE anti-FOXP3 (eBioscience) antibodies or isotype controls, and FACS analysis was performed as previously described [58].

### Statistical analysis

 The results were expressed as means ± standard deviation. *P* values ≤ 0.05 were considered significant. One-way analysis of variance (ANOVA), Chi-square tests and Mann-Whitney tests were used when appropriate. To detect correlations between serum NT levels, clinical and other biological data, linear regression analysis was used and *p*-values were determined by Spearman’s rank correlation test.

## Results

### Variations in serum NT expression in SLE

#### The NT levels in sera of SLE patients, NGF, BDNF and NT-3 were determined by ELISA

**Figure 1:**
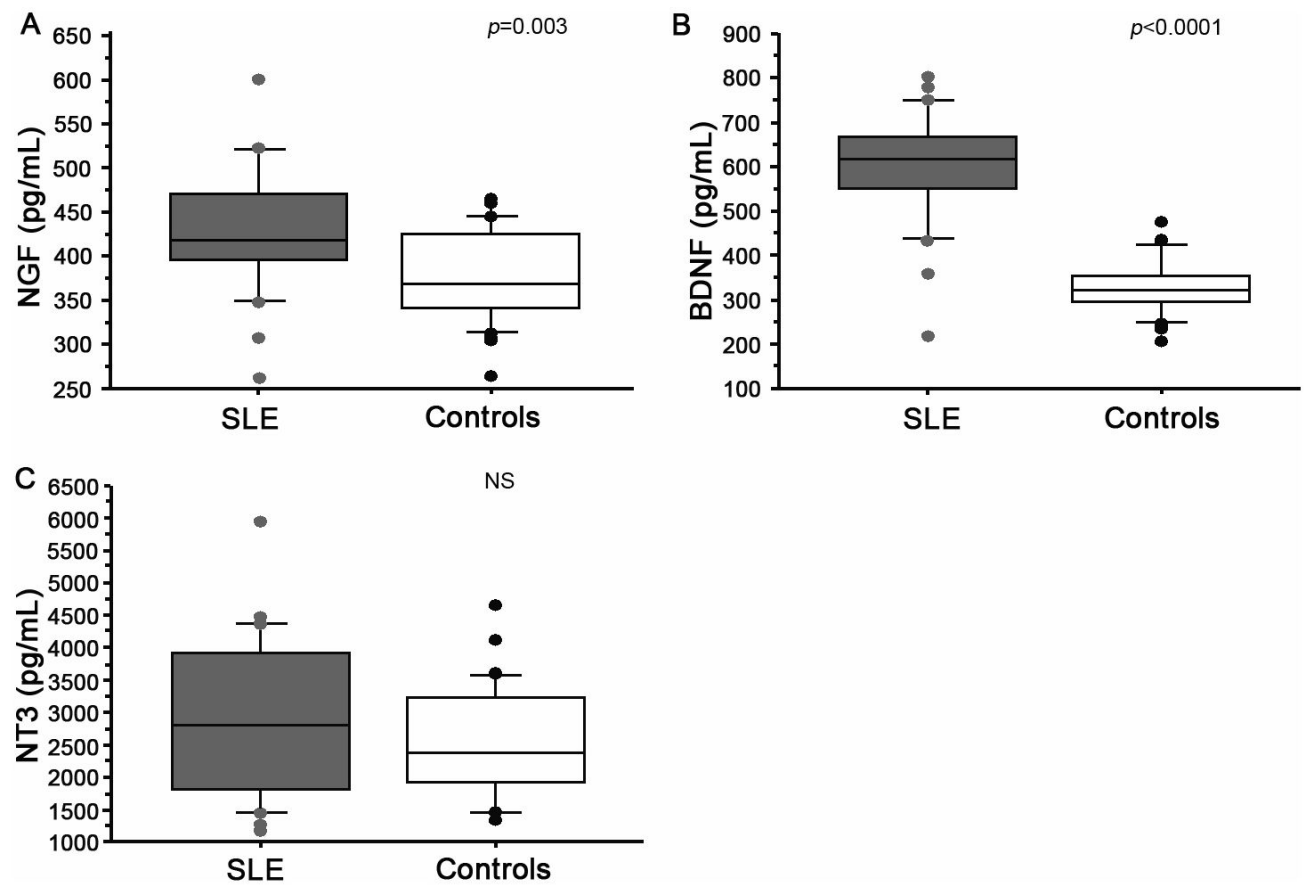
Concentrations of serum NGF (A), BDNF (B) and NT-3 (C) detected by ELISA in SLE patients (SLE, grey boxes) and healthy controls (Controls, white boxes). The boxes represent the 50th percentile, the bars outside the boxes show the 10th and 90th percentiles, and the horizontal black lines represent the median. Significant differences were assessed with Mann-Whitney tests.

Serum NGF levels were higher in SLE patients (426.13 ± 70.85 pg/mL) than in healthy controls (373.9 ± 52.3 pg/mL, *p*=0.003, Figure 1A). BDNF levels were also increased in SLE patients (598.9 ± 129.8 *vs* 326.1 ± 60.5 pg/mL in controls, *p*<0.0001, Figure 1B). Average serum NT-3 levels were similar in the SLE group and controls (2911.7 ± 1248.8 *vs* 2553.7 ± 879.7 pg/mL, NS, Figure 1C).

The increases in serum levels of NGF and BDNF were statistically independent (Figure 1D, r=0.28, NS). Therefore, we examined the correlation between enhanced NGF and BDNF levels and lupus systemic activity.

### Serum neurotrophins and SLE activation profile

**Figure 2:**
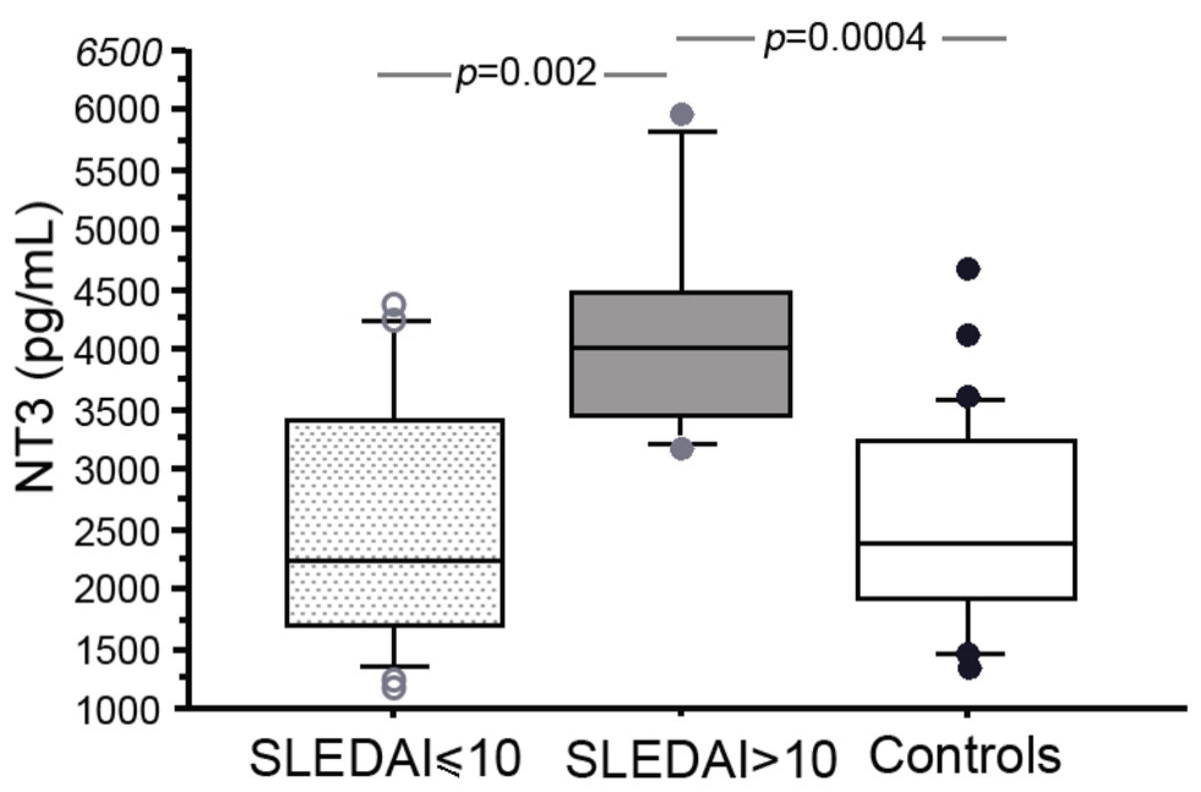
Serum NT-3 concentrations (ELISA) in SLE patient with severe systemic flare (SLEDAI>10, grey box), moderate flare (SLEDAI≤10, dotted box) and healthy controls (Controls, white box). The boxes represent the 50th percentile, the bars outside the boxes show the 10th and 90th percentiles, and the horizontal black lines represent the median. Significant differences were assessed with Mann-Whitney tests.

NGF, BDNF and NT-3 serum levels were not correlated with initial SLEDAI score (NGF: Γ=0.19, *p*=0.34, BDNF: Γ=0.16, *p*=0.42, NT-3: Γ=0.35, *p*=0.07). However, there were higher NT-3 levels in a subset of patients with severe systemic flare (SLEDAI ≥10; 4171.6 ± 1013.17 *vs* 2533.7 ± 1062.9 pg/mL, *p*=0.002, Figure 2). In contrast, concentrations of NGF (426.2 ± 62.6 *vs* 425.9 ± 74.6 pg/mL, NS) and BDNF (612.6 ± 73.6 *vs* 594.7 ± 143.7 pg/mL, NS) were similar in patients regardless of SLEDAI score.

SLEDAI score reflects the global systemic activity of SLE, which can correspond to the involvement of various organs. The presence of cutaneous, neurological or renal SLE manifestations did not influence serum levels of NGF, BDNF and NT-3 (Table 1). However, NT-3 levels were significantly increased in patients with articular manifestations (3185.6 ± 1213.3 pg/mL *vs* 1761.7 ± 581.2 pg/mL, *p*=0.02). In contrast, there was no difference in NGF and BDNF levels between patients with and without joint involvement (Table 1). 

**Figure 3:**
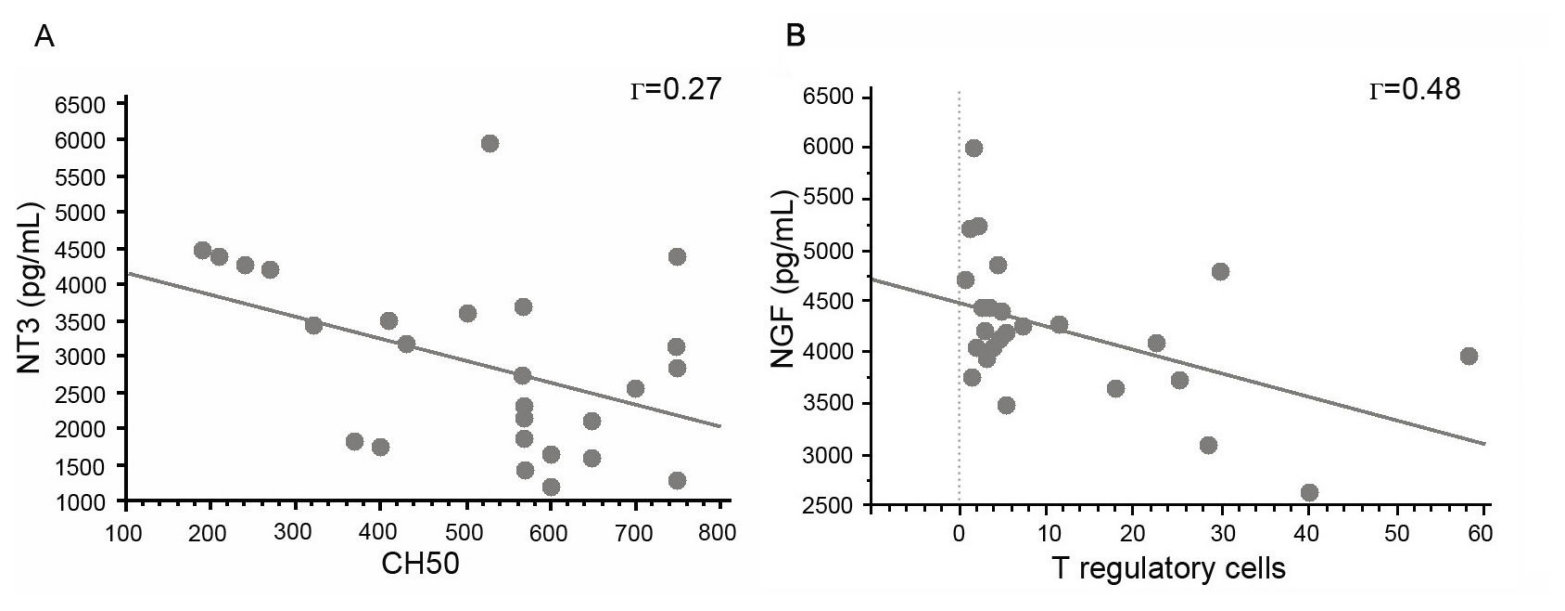
Correlations between NT-3 and CH 50 NGF (A) and T regulatory cell counts (flow cytometry) in SLE patients (B). The linear regression curve and coefficient of determination yielded by the analysis of variance are both represented. Each point represents an individual patient and a healthy control. Γ defines the coefficient of determination yielded by the analysis of the variance table. *P*-values were determined by Spearman’s rank correlation test.

We then investigated the correlation between NT serum levels and immunological parameters associated with lupus flare. Serum NT-3 levels alone were dramatically increased in patients with complement activation (n=8, 3749.1 ± 1433.25 *vs* 2457.9 ± 950.7, *p*=0.01) and these levels correlated negatively with serum CH 50 levels (Γ=-0.28, *p*=0.032, Figure 3A, Table 1). In contrast, anti-nDNA antibodies are independent of the serum levels of NGF (Γ=0.09, *p*=0.68), BDNF (Γ=0.29, *p*=0.18) and NT-3 (Γ=0.35, *p*=0.07).

Considering that SLE may be associated with secondary Sjögren syndrome or anti-phospholipid syndrome, we examined if there was a correlation between NT serum levels and SLE-associated autoimmune disease.

### Serum neurotrophins and SLE-associated disease

NGF, BDNF and NT-3 concentrations were similar in SLE patients with or without secondary Sjögren’s syndrome (NGF 430.7 ± 113.2 *vs* 424.5 ± 51.8 pg/mL, BDNF 624.27 ± 102.31 *vs* 589.4 ± 139.89 pg/mL, NT-3 3094.86 ± 1262.33 *vs* 2860.87 ± 1274.52 pg/mL, all NS). However, the presence of anti-SSB antibodies (n=3) was associated with higher serum NGF levels (507.2 ± 23.4 *vs* 417.4 ± 69.1, *p*=0.03, Table 1).

In patients with anti-phospholipid autoantibodies, lower serum BDNF levels were observed: anti-cardiolipin (n=9, 529.2 ± 142.9 *vs* 635.7 ± 109.2 pg/mL, *p*=0.04) or anti-β2GP1 antibodies (n=4, 438.8 ± 185.4 *vs* 627.9 ± 96.87 pg/mL, *p*=0.004). Furthermore, BDNF levels negatively correlated with the titers of both IgG and IgM anti-cardiolipin antibodies (Γ=0.46, *p*=0.01 and Γ=0.41, *p*=0.04, respectively, Table 1). 

In addition, we examined a possible correlation between systemic flare, quality of life and serum NT profile.

### Serum neurotrophins and quality of life

 Scores from both the Physical Component Summary (PCS) and Mental Component Summary (MCS) were lower in SLE patients than healthy controls (PCS, 36.5 ± 6.9 *vs* 54.1 ± 7.4, *p*<0.0001; MCS, 38.6 ± 10.1 *vs* 50.2 ± 6.5, *p*<0.0001) and did not correlated with SLEDAI score (PCS: Γ=0.03, *p*=0.87, MCS: Γ=0.22, *p*=0.32). In SLE patients and the controls, neither PCS nor MCS scores were related to serum NT levels.

*In order to evaluate the influence of immunomodulating drugs on the increased circulating levels of NT, we examined systemic NT levels before and after SLE flare treatment.*

### Dynamics of neurotrophin serum profile and SLE treatment

Serum NGF, BDNF, and NT-3 concentrations were similar in SLE patients at the time of the blood sample whether they were untreated (n=4) or previously treated by corticosteroid, hydroxychloroquine or immunosuppressants (Table 1).

**Figure 4:**
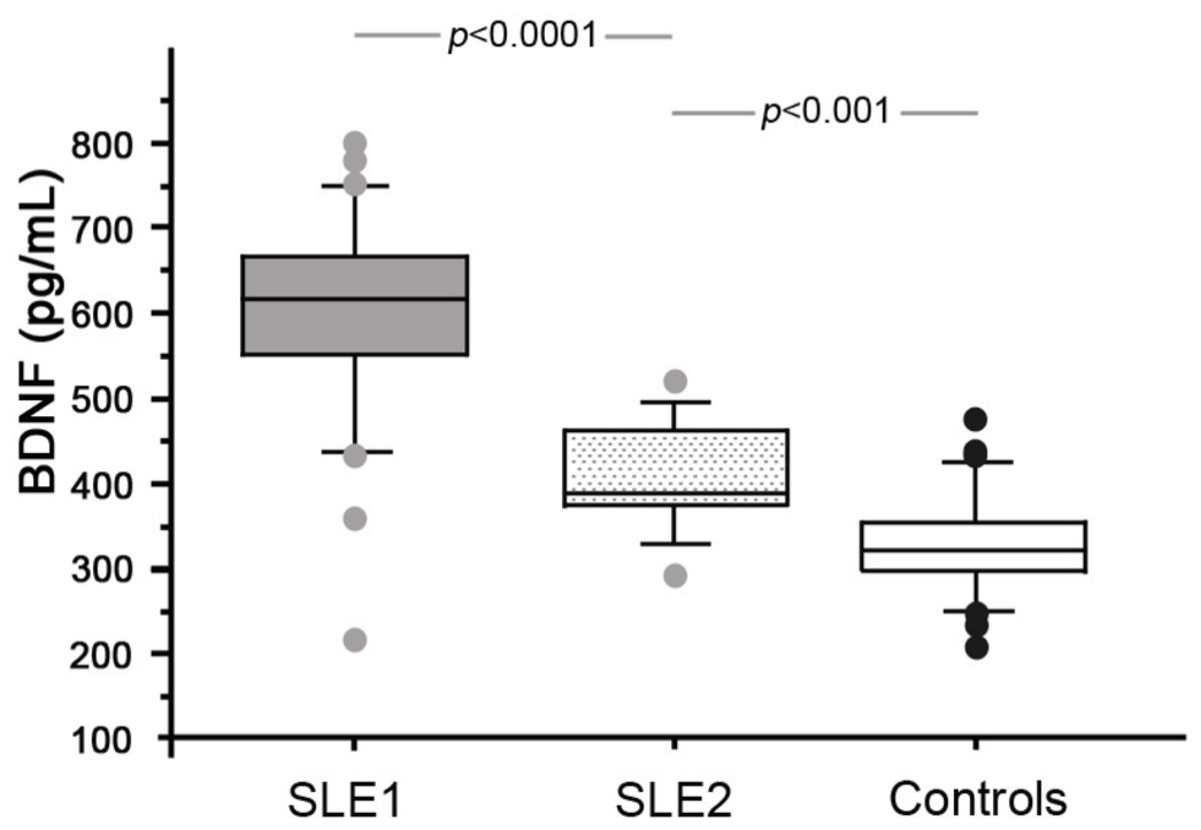
Serum BDNF concentrations in SLE patients before (SLE1, grey box) and after (SLE2, dotted box) treatment of the disease flare compared to healthy controls (Controls, white box). The boxes represent the 50th percentile, the bars outside the boxes show the 10th and 90th percentiles, and the horizontal black lines represent the median. Significant differences were assessed with Mann-Whitney tests.

Interestingly, serum BDNF concentrations decreased one month after the systemic flare of the disease (407.7 ± 65.6 pg/mL) but were still higher than those in healthy volunteers (326.1 ± 60.5 pg/mL, *p*<0.001, Figure 4). In contrast, serum levels of NGF (426.12 ± 70.8 *vs* 462.6 ± 47.1 pg/mL, NS) and NT-3 (2986.9 ± 954.5 *vs* 2911.7 ± 1248.8 pg/mL, NS) remained unchanged after treatment of the SLE flare. NGF, BDNF and NT-3 levels remained uncorrelated with the SLEDAI score (mean 4 ± 1.8) assessed after flare treatment (Γ=0.35, Γ=0.46 and Γ=0.27 respectively, all NS).

 In order to determine if there was a correlation between NT and SLE activity at the level of immune cells, we tested if there was a relationship between serum and lymphocytic NT expression, T and B cell activation, and cytokine profiles.

### Lymphocytic neurotrophins and clinical and immunological SLE profile

#### NGF and BDNF-producing B cells are increased in SLE

**Figure 5:**
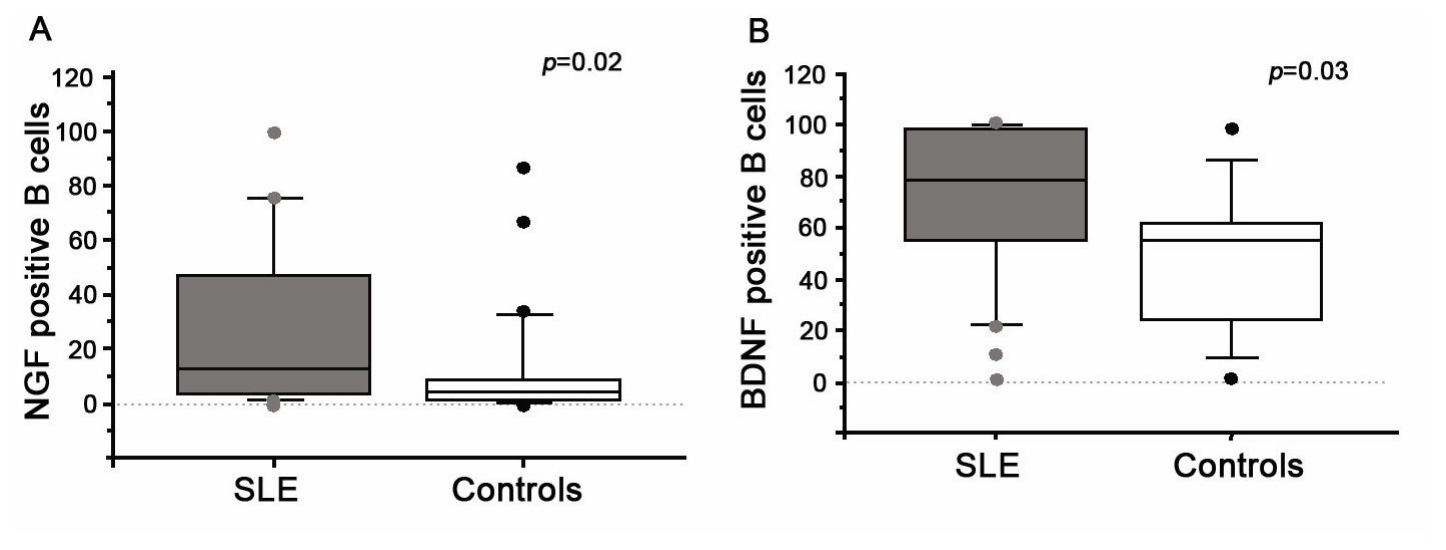
Percentage of B lymphocytes (flow cytometry) expressing NGF (A) and BDNF (B) (SLE, grey boxes) and healthy controls (Controls, white boxes). The boxes represent the 50th percentile, the bars outside the boxes show the 10th and 90th percentiles, and the horizontal black lines represent median values. Significant differences were assessed with Mann-Whitney tests.

The numbers of B cells producing NGF and BDF were greater in the SLE group than in healthy controls (NGF, 29.3 ± 31.3 *vs* 11.3 ± 20.9, *p*=0.02; BDNF, 71.2 ± 30.9 *vs* 47.9 ± 27.8, *p*=0.03, Figure 5). Cell numbers were independent of serum NGF, BDNF and NT-3 concentrations. In contrast, the numbers of NT-3-CD19-positive cells were similar in the two groups (72.4 ± 29.7 *vs* 64.7 ± 29.1, NS).

The numbers of NGF and BDNF-producing B cells were independent of SLEDAI score (Γ=0.22 and Γ=0.2 respectively, NS). Although numbers of NGF-producing cells were independent of clinical SLE profiles, CD19+ BDNF-producing B cells were dramatically decreased in patients with an associated anti-phospholipid syndrome (40.2 ± 8.9 *vs* 80.5 ± 22.05, *p*=0.03). 

NGF and BDNF-producing B cells were not influenced by corticosteroid (NGF: 36.5 ± 33.8 *vs* 20.8 ± 27.11, NS, BDNF: 75.4 ± 23.7 *vs* 88.8 ± 17.5, NS), hydroxychloroquine (NGF: 33.6 ± 23.6 *vs* 14.7 ± 16.8 NS, BDNF: 79.9 ± 23.3 *vs* 84.3 ± 19.2, NS) or immunosuppressants (NGF: 25.3 ± 19.8 *vs* 30.1 ± 33.3, NS, BDNF: 76.06 ± 30.2 *vs* 81.6 ± 21.7, NS). 

Interestingly, NGF production by B cells tended to be higher in SLE patients with cryoglobulinemia (mean fluorescence intensity, Xmean, 8.7 ± 7.7) than in patients without cryoglobulinemia (Xmean 4.1 ± 2.7, *p*=0.06). A similar relationship was observed between CD19-related NGF production and complement activation (Xmean 7.1 ± 5.41 *vs* 3.5 ± 1.8, *p*=0.05). 

CD19-BDNF-producing B cells were also decreased in patients positive for lupus anticoagulants (28.5 ± 14.3 *vs* 79.0 ± 23.9, *p*=0.01).

#### NGF and BDNF-producing T cells in SLE

The numbers of T lymphocytes expressing NGF (17.2 ± 21.2 *vs* 9.8 ± 21.3, NS), BDNF (55.1 ± 33.5 *vs* 55.1 ± 33.5, NS) and NT-3 (57.5 ± 33.7 *vs* 52.9 ± 24.2, NS) were similar in the SLE and control groups and were independent of patients’ clinical and immunological SLE profiles.

#### NT and B-cell activation parameters

As expected, serum BAFF levels were increased in the SLE group (1980.7 ± 1315.9 *vs* 1019.9 ± 193.4 pg/mL, *p*=0.01). However, they did not correlate with serum concentrations of NGF, BDNF, or NT-3 (Γ=0.12, Γ=0.03 and Γ=0.04, respectively) in the SLE group. 

In contrast, in the control group, serum concentrations of NGF positively correlated with BAFF levels (Γ=0.64, *p*=0.02), which were also independent of the serum levels of both BDNF and NT-3 (Γ=0.02 and Γ=0.6, respectively). 

Serum levels of BAFF were also independent of B and T cell NT expression in both the SLE and control groups. 

#### NT and T regulatory cells

Only serum NGF levels and T regulatory cell counts were negatively correlated in SLE patients (Γ=0.48, *p*=0.01, Figure 3B), whereas there was no significant correlation between BDNF or NT-3 levels and T regulatory cell numbers (Γ=0.06, NS, Γ=0.28, NS, respectively). T regulatory cells and NTs appeared to be independent in healthy controls (NGF Γ=0.27, BDNF Γ=0.12, NT3 Γ=0.08, NS).

 BDNF-positive CD19 cells and T regulatory cells were negatively correlated (Γ=0.39, *p*=0.04).

#### NT and IL-10 production

IL-10 concentrations were higher in the SLE group (29.4 ± 81.1 *vs* 0.19 ± 3.3 pg/mL, *p*=0.06), especially in the subgroup of patients with severe systemic flare (91.28 ± 162.6 *vs* 10.9 ± 11.26 pg/mL, *p*=0.03) or positive anti-SSA antibodies (77.25 ± 152.48 *vs* 12.24 ± 13.27 pg/mL, p=0.07).

In the SLE group, NGF, BDNF and NT-3 serum levels were statistically independent of serum IL-10 levels (Γ=0.02, Γ=0.12 and Γ=0.12 respectively), which did not influence NT production in T or B cells. The same results were observed in the control group.

#### NT and Interferon-*γ* production

Serum INF-γ levels were higher in the SLE group (INF-γ 136.7 ± 230.9 *vs* 6.9 ± 10.5 pg/mL, *p*=0.03), particularly in patients with severe systemic flare (258.9 ± 422.32 *vs* 79.58 ± 98 pg/mL, *p*=0.08). NGF, BDNF and NT-3 levels were also statistically independent of INF-γ concentrations (Γ=0.21, Γ=0.17 and Γ=0.35, respectively) in the SLE group. In contrast, serum BDNF levels negatively correlated with INF-γ concentration in the control group sera (Γ=0.55, *p*=0.03).

While NT production by B and T cells was independent of INF-γ concentration in the control group, INF-γ serum levels correlated with NGF production in T lymphocytes (Γ=0.64, *p*=0.01). 

## Discussion

 The present study provides new evidence of the significant variation in NT levels in both serum and circulating B lymphocytes in SLE patients. Previous studies mainly focused on serum NGF levels, and serum BDNF and NT-3 had not been studied in a SLE cohort. Furthermore, NT expression in circulating B cells had not yet been documented. We have identified an increase in NGF and BDNF levels in both serum and circulating blood CD19-B cells in patients. Interestingly, NT-3 levels were only increased in severe forms of SLE and correlated with complement activation. 

 We have identified a significant increase in sera NGF and BDNF levels in SLE patients compared to a healthy control group. We confirm that serum NGF levels are increased in SLE patients, a result previously demonstrated [43]. However, we did not find any correlation between serum NGF levels, systemic complications, and SLEDAI scores in this SLE population, characterized by previous treatment with either corticosteroid or immunosuppressants. These results differ from those of a previous study reporting a correlation between serum NGF levels and SLE activity [44]. However, the inclusion criteria of this study was different, involving only untreated children (and not adults) with an over-representation of renal involvement (60% of patients *vs* 16% in the present study) [44]. Moreover, a link between renal involvement and enhanced NGF levels has been described in SLE mice and in patients with SLE glomeronephritis-related renal insufficiency [42,59], which could explain this discrepancy. However, we did not find any correlation between NGF serum levels and the presence of renal involvement (only 4 adults) or other systemic complications. Therefore, the effect of immunomodulating drugs on serum NGF levels in previously treated patients needs to be discussed in regards to other reports. In fact, NGF serum levels have been found to be independent of immunomodulating treatments in other diseases such as systemic sclerosis, rheumatoid arthritis, and primary Sjögren syndrome [36,38,60]. 

Concerning BDNF levels, this study reports for the first time an increase in serum BDNF levels in SLE patients, occurring independently of central neurological involvement (absent in all but two patients). Serum BDNF levels did not correlate with SLEDAI score. Only one previous case report has been described of psychotic involvement in SLE with an increase of serum BDNF [61]. Likewise, in multiple sclerosis and rheumatoid arthritis, a decrease in serum BDNF levels occurs with clinical improvement, independently of other inflammatory markers [62,63]. After treatment, a decrease in BDNF levels is detected in these SLE patients. The significance of this decrease with treatment is debated and also appears to be independent of both corticosteroid and immunosuppressive drugs in psychiatric form of SLE patients [46], as well as in systemic sclerosis [36] and rheumatoid arthritis [60]. In contrast, BDNF serum levels are enhanced in primary Sjögren syndrome patients (pSS) treated with either corticosteroid or immunosuppressants for severe systemic involvement [38]. Interestingly, we have shown that BDNF levels decrease after treatment of systemic flares, signifying that this decrease could be a biological marker for improvement and a therapeutic response in SLE [46]. 

Strikingly, we find an increase of NT-3 serum levels only in severe (SLEDAI ≥ 10) and articular forms of SLE. Serum NT-3 has been infrequently studied in inflammatory diseases, although NT-3 immune function has been demonstrated in asthma: autocrine NT3 autocrine secretion leads to plasma cell survival [4]. NT-3 involvement in autoimmune articular symptoms is indicated by its presence in the synovial fluid of patients with spondyloarthritis [64] and by increased levels in the sera of systemic sclerosis patients with articular complications that require hydroxychloroquine treatment [36]. Enhanced NT-3 serum levels in severe forms of SLE could reflect a link between NT-3 and lupus flare, in that complement activation (decrease of CH 50 levels) correlates with elevated NT-3 levels. This direct relationship between NT-3 synthesis and complement (C1q) has been shown in neuronal cells [65]. In another cell model, NT-3 production by endothelial cells is upregulated by local ischemia [66]. Therefore, NT-3 secretion could be a biological marker of severe SLE forms, potentially associated with vascular damage related to an active vasculitis process. 

 The direct impact of NT on SLE physiopathology is also underlined by the significant overproduction of both NGF and BDNF by B cells, and could reflect an activation of circulating B cells in SLE. The activation of circulating B-cells in SLE patients has been reported during nephritis and SLE activity [67]. Moreover, in some experimental conditions, a direct link between *in vitro* activation of B cells and their secretion of NGF and BDNF has been established [2,7-10,13]. Overall, the overexpression of these NTs in SLE patients’ circulating B cells (compared to healthy controls) could be a hallmark of activation that correlates with the disease. 

To further evaluate the impact of NTs on SLE pathogenesis, we have looked for correlations between both serum and lymphocytic NT levels and immune characteristics, especially antibody production and T regulatory cell profiles. 

NGF, BDNF and NT-3 levels did not correlate with anti-nDNA levels. This correlation has not been previously examined in the 3 published studies on NGF and BDNF serum levels in SLE [43,44,46]. This absence of relationship between NT serum levels and autoantibody production was also found in pSS and systemic sclerosis [13,37]. Concerning rheumatoid arthritis, to our knowledge, the correlation between NGF, BDNF and anti-cyclic citrullinated peptide has not been previously studied [60,64].

Interestingly, serum BDNF levels were the lowest in the group positive for anti-phospholipid. This negative correlation could be due to anti-phospholipid-associated vasculopathy and oxidative stress, an association previously identified in systemic sclerosis, atherosclerosis and diabetes [36,68,69]. Similarly, we identified for the first time a striking decrease of BDNF-producing B cells in patients with an associated anti-phospholipid syndrome. These results suggest that a reduction of both circulating BDNF levels and BDNF-producing B cells are involved in the vascular damage associated with SLE. 

 In addition, we report a negative correlation between serum NGF levels and T regulatory cells in SLE patients, which could reflect either SLE activity [70] or a direct impact of serum NGF level on T regulatory cell survival. Also, NGF is an inducible survival growth factor for T cells, depending on the cytokine profile [26]; anti-NGF treatment enhances Foxp3+ regulatory T cells and decreases Th17 cells in a murine model of asthma [71]. Moreover, IL-17 enhances NGF production in human T cells [72]. Together, this data suggests that NGF could regulate the balance of T-regulatory and Th17 cells [26,71]. Nevertheless, no reports have shown that either p75^NTR^ or TrkA NGF receptors are present on human T regulatory cells, which are known to produce high levels of BDNF in HIV-associated neurodegeneration models [73]. 

IL-10 serum levels were enhanced in the SLE group, reflecting systemic activity and anti-SSA production as previously described [48-51], whereas NT and IL-10 levels are were not correlated. Indeed, IL-10 release by immune cells, which is enhanced by both NGF and BDNF in normal conditions, is dramatically reduced in cases of allergy [74].

As expected, INF-γ serum levels were increased in SLE. Interestingly, in our control group only, we found a negative correlation between serum BDNF level and INF-γ, which is known to reduce BDNF production in neurons, glia, and bronchial smooth muscle cells [21,75]. Moreover, a cytokine balance between INF-γ and BDNF has been described in bowel mucosa, as a regulating factor of enteric glia cell apoptosis [30]. The deregulation of the INF-γ and BDNF balance in SLE may reinforce the importance of BDNF over-secretion in SLE pathogenicity.

In conclusion, the present study finds that the expression of the neurotrophins NGF and BDNF, overexpressed by circulating B cells, are increased in the sera of SLE patients independently of Th1/Th2 profile. NT-3 is upregulated only in severe flares of the disease, and BDNF levels are closely related to anti-phospholipid syndrome. 

### Thus, we hypothesize that evaluating NT in both sera and circulating-B lymphocytes could be a new biological marker of SLE activity and systemic complications

 To test this hypothesis, prospective large cohort studies, including naive SLE patients, need to be performed. In addition, further studies of NT-secreting subpopulations of B and T cells, in association with their NT receptors (i.e. TrkA, TrkB, TrkC, p75^NTR^ and sortilin), will be conducted to define their fine-tuning functions during SLE disease. This study supports the idea that neurotrophins are involved in SLE physiopathology and thus could be a potential target of systemic treatment.
